# Global research trends and focus on the link between rheumatoid arthritis and neutrophil extracellular traps: a bibliometric analysis from 1985 to 2023

**DOI:** 10.3389/fimmu.2023.1205445

**Published:** 2023-08-23

**Authors:** Yonglong Chang, Qinling Ou, Xuhui Zhou, Kechao Nie, Jinhui Liu, Sifang Zhang

**Affiliations:** ^1^ Department of Integrated Traditional Chinese & Western Medicine, The Second Xiangya Hospital, Central South University, Changsha, China; ^2^ National Clinical Research Center for Metabolic Diseases, Changsha, China; ^3^ Department of Addiction Medicine, Hunan Institute of Mental Health, Brain Hospital of Hunan Province (The Second People’s Hospital of Hunan Province), Changsha, Hunan, China; ^4^ College of Integrated Traditional Chinese & Western Medicine, Hunan University of Traditional Chinese Medicine, Changsha, Hunan, China

**Keywords:** neutrophil extracellular traps, rheumatoid arthritis, bibliometric analysis, research trends, Citespace, VOSviewer

## Abstract

Rheumatoid arthritis (RA) is an autoimmune disease that currently has an unknown cause and pathogenesis, and is associated with many complications and a high disability rate. The neutrophil extracellular trap network (NETs) is a newly discovered mechanism that allows neutrophils to capture and kill pathogens. Multiple studies in recent years have highlighted its relevance to the progression of rheumatoid arthritis. Despite the growing number of studies indicating the crucial role of NETs in RA, there has been no bibliometric review of research hotspots and trends in this area. In this study, we retrieved articles related to NETs in RA from the Web of Science Core Collection (WoSCC) database from 1985 to 2023 and used visualization tools such as Citespace, VOSviewer, Tableau Public, and Microsoft Office Excel 2021 to analyze the data. After screening, we included a total of 416 publications involving 2,334 researchers from 1,357 institutions in 167 countries/regions, with relevant articles published in 219 journals. The U.S., China, and Germany are the top 3 countries/regions with 124, 57, and 37 publications respectively. Mariana J. Kaplan is the most published author, and journals such as Frontiers in Immunology and International Journal of Molecular Sciences have had a significant impact on research in this field. The clinical application of PAD enzymes and their inhibitors, and the drug development of NETs as therapeutic targets for RA is a trend for future research. Our study provides a comprehensive bibliometric analysis and summary of NETs in RA publications, which will aid researchers in conducting further scientific research.

## Introduction

Rheumatoid arthritis (RA) is an autoimmune disease characterized by chronic inflammation that mainly affects middle-aged and elderly women (1). It usually presents progressive, symmetric, polyarticular nonsuppurative inflammation (2). Studies have shown that the global prevalence of rheumatoid arthritis is between 0.1% and 0.9% (3). Although the pathogenesis of RA is not yet fully understood, in the past few years, a growing body of research has highlighted the critical role that neutrophil extracellular trapping networks (NETs) may play in the pathogenesis of RA (4–6).

Neutrophils are an essential part of the innate immune system and serve as the first line of defense against invading pathogens (7). In addition to their role in fighting infections, they also contribute to the immune response in cancer and autoimmune diseases. In 2004, Brinkmann et al. (8). discovered that neutrophils can undergo a process known as NETosis (9) wherein they extrude their DNA backbone with a reticulated fibrous structure of granular proteins and peptides attached to it, producing a structure called NETs. NETs have two pathways of production, depending on whether it relies on nicotinamide adenine dinucleotide phosphate (NADPH) or not (10)While NETs play an important role in host defense against bacterial infections, their over-activation can cause tissue damage, making them a “double-edged sword.” NETs are closely associated with many inflammatory and autoimmune diseases, including RA.

Research on the role of NETs in RA is still in its early stages, but there have been important advances. Studies have demonstrated that RA patients’ neutrophils release more NETs, which may contribute to the persistence of inflammation and joint damage (11). NETs can affect the immunopathology of RA in various ways. Moreover, research on drugs that inhibit NETs formation and activity, such as PAD inhibitors and DNA enzymes, has shown promise in animal models (12). Additionally, measuring the levels of NETs in patients’ blood can be used as a biomarker to assess the severity and prognosis of RA and guide treatment options (13). However, the findings have not been systematically consistent, and there is currently no bibliometric focus on the current status and trends of research on NETs in RA.

Bibliometrics is an interdisciplinary field that emerged in 1969 (14), which combines mathematics, statistics, and bibliography to statistically analyze and visualize literature, authors, institutions, and countries/regions to assess current research status and trends (15). In this study, we utilize bibliometric methods to investigate changes in the research structure and future research trends of NETs in RA over the past four decades. Our objective is to build a scientific knowledge map of this field and provide valuable insights for future research in this area (16).

## Methods

### Data source and search strategy

**Figure 1:**
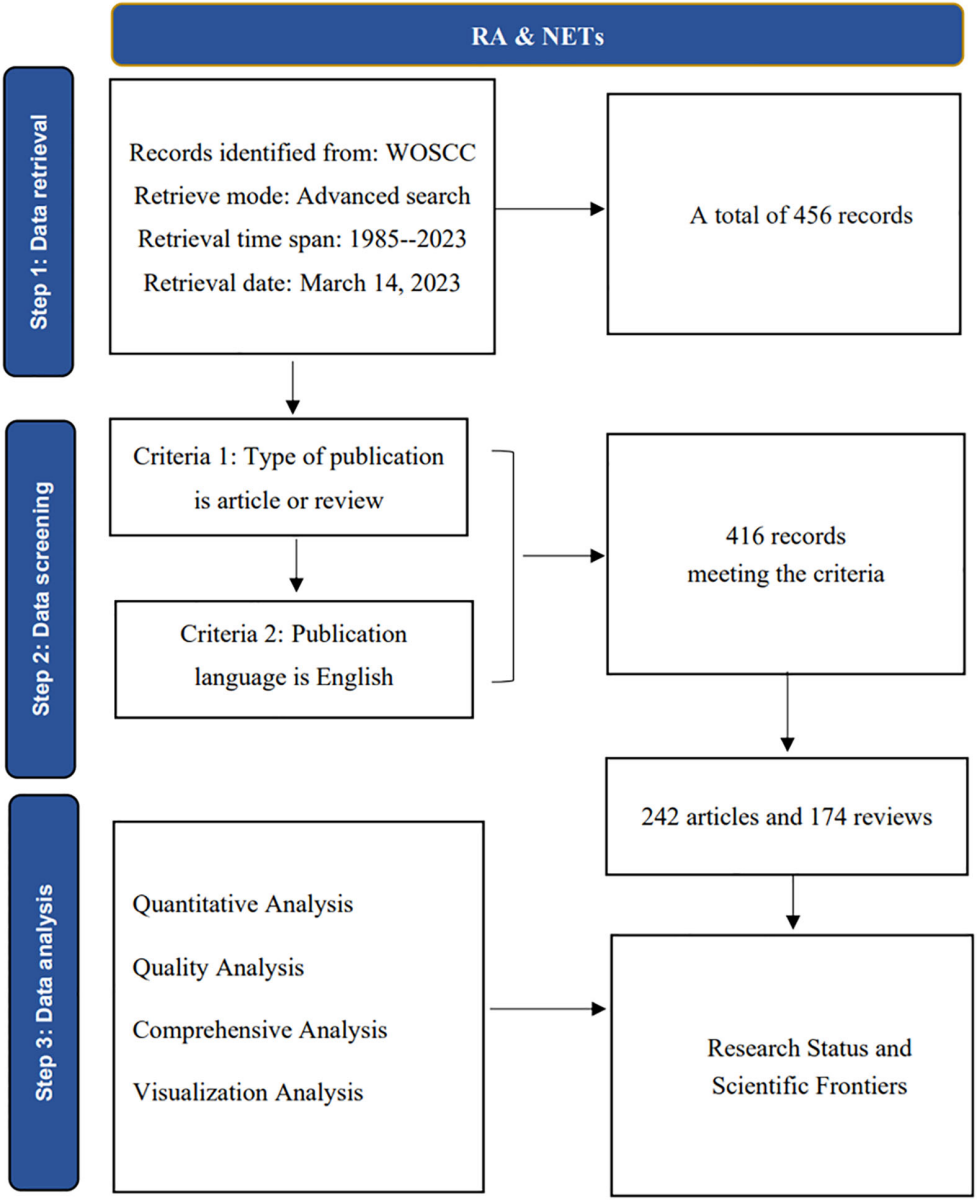
Flow chart for inclusion and exclusion of publications.

To obtain data for this study, we used the Web of Science Core Collection (WoSCC) database, which can be accessed at: https://www.webofscience.com/wos/woscc/basic-search. To ensure consistency in our data, we downloaded all relevant information on March 14, 2023, and applied the search notation TS= ((“Neutrophil Extracellular Traps” OR “NETs” OR “NETosis”) and (“Rheumatoid arthritis” OR “RA”)), covering the period from 1985-01-01 to 2023-03-14. This search yielded a total of 456 publication records, which were then screened by two staff members using specific inclusion and exclusion criteria (**Figure 1**). After this process, we obtained 416 valid publications. We saved information related to the titles, authors, keywords, citations, journals, institutions, and references of these publications in plain text format (17).

### Data analysis

The literature dosage analysis software CiteSpace [version 6.1], VOSviewer [version 1.6.18], and the website https://bibliometric.com/app were used to analyze the overview of the development of NETs in RA over the last 40 years, trends and new frontier hotspots, including country/region, institution, journal distribution, and keywords.

CiteSpace [version 6.1] is a software tool that analyzes co-authorship networks of authors and institutions using co-occurrence analysis. The resulting graphs depict a network of nodes, where each node represents an author or institution, and the size of the node corresponds to its prominence in the network. The connections between nodes indicate co-occurrence or co-citation relationships, and the thickness of the connection reflects the strength of the collaboration. In other words, the more interconnections between nodes, the thicker the connection and the stronger the co-occurrence or co-citation relationship. Overall, CiteSpace [version 6.1] provides a visual representation of the co-authorship network and can be used to identify key authors or institutions within a particular field of research.

VOSviewer [version 1.6.18] was used to visualize the co-occurrence and co-citation patterns among references, journals, authors, and keywords. In the resulting co-citation map, each element is represented by a point, with the size of the point indicating the number of publications or citations associated with it. The lines connecting the points show co-citation relationships between the elements. The dots and lines are color-coded to indicate different clusters or years.

The geographical distribution of NETs publications in RA is created using Tableau Public [version 2021.3], which is a data visualization tool that allows users to create interactive charts and maps. Meanwhile, Microsoft Office Excel 2021 is utilized for conducting quantitative analysis of the publications.

## Results

### Annual trends in publications

**Figure 2:**
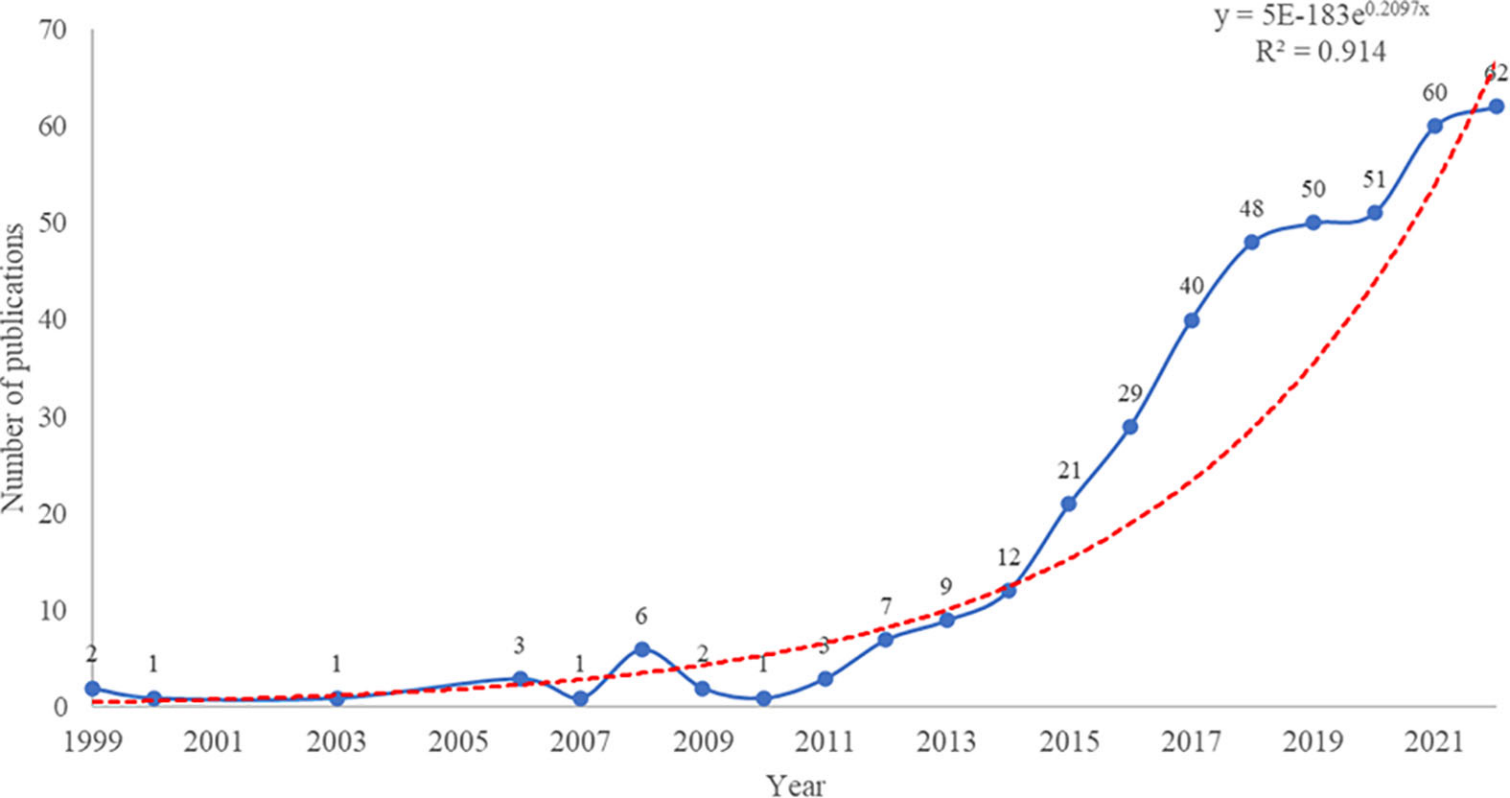
Worldwide publication growth trends over the period 1985-2022.

The study analyzed 416 publications on NETs in RA, authored by 2,334 researchers from 1,357 institutions in 167 countries/regions, and published in 219 journals. **Figure 2** illustrates the publication trend over the last 40 years, which reveals a significant surge in the annual volume of publications in this field after 2015. The publication rate has remained high, with over 50 publications per year since 2019. Additionally, the polynomial curve shows a positive correlation between the number of annual publications and the year of publication (R^2^ = 0.914) (18, 19). These findings indicate that NETs have gained increasing attention from scholars in recent years and are becoming a significant focus of RA research.

### Analysis of countries and institutional

**Figure 3:**
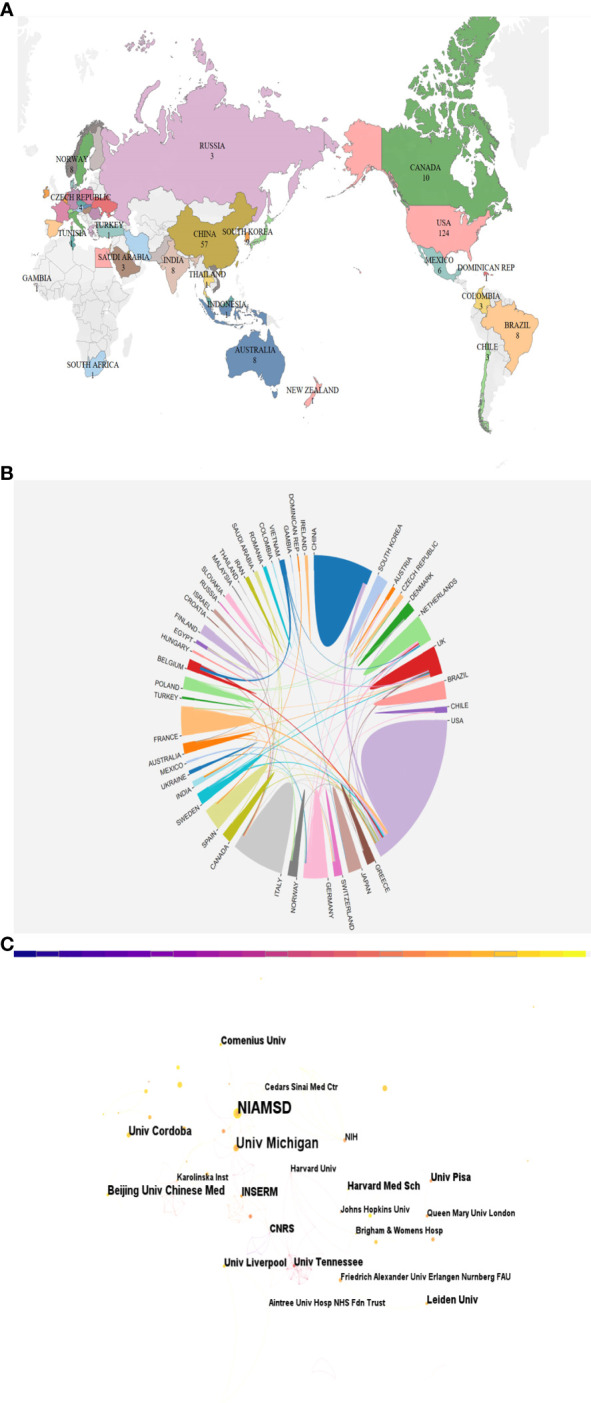
**(A)** A map depicting the geographic distribution of publications across various countries/regions based on their total number. **(B)** Map of inter-country/inter-regional publication cooperation. **(C)** Visualization of institutions associated with NETs in RA publications.

Over 100 countries and regions have made significant contributions to research on NETS in RA. The distribution and representation of these countries can be visualized in **Figure 3A**, while **Table 1** outlines the top 10 countries and institutions in terms of publication volume. The US ranks first with 124 publications, followed by China with 57, Germany with 37, Italy with 32, and the UK with 35, among others. Notably, the top 10 countries in terms of publication volume are spread across North America, Europe, and Asia.

**Table 1:** Top 10 active countries/regions and institutions.

Rank	Country	Records	Institutions	Records
1	USA	124	NIAMS	17
2	CHINA	57	University of Michigan	13
3	GERMANY	37	Beijing university of Chinese Medicine	7
4	ITALY	36	University of Córdoba	7
5	ENGLAND	35	INSERM	6
6	NETHERLANDS	27	University of Liverpool	5
7	FRANCE	21	Leiden University	5
8	SPAIN	20	Harvard Medical School	5
9	JAPAN	20	University of Pisa	5
10	SWEDEN	14	University of Tennessee	5

Regarding international cooperation in this field, several countries have established partnerships as depicted in **Figure 3B**. The data suggests a positive correlation between the number of publications by a country/region and the degree of collaboration. The United States, with the highest number of publications, has notably close partnerships with China and France. Additionally, China and Germany have established the most extensive collaborations with other countries, apart from the US.

**Figure 3C** depicts the number of publications and collaborative relationships among institutions. The National Institute of Arthritis and Musculoskeletal and Skin Diseases (NIAMS) has contributed the highest number of publications, with 17 publications in this area. Among the top ten institutions in terms of publication volume, many are based in the US, China, France, Argentina, the Netherlands, and Italy.

### Analysis of top journals and co-cited journals

A total of 219 journals have published studies related to NETs in RA. Among them, Frontiers in Immunology was the most commonly published journal with 46 articles, followed by International Journal of Molecular Sciences and Autoimmunity Reviews, each with 13 articles. Arthritis & Rheumatology published 11 articles, while Journal of Immunology had 9 articles on this topic. The top 10 journals that published studies related to NETs in RA had an average impact factor of 8.982 and the highest impact factor in 2023 was 17.39, indicating a strong influence in the scientific community. Please refer to **Table 2** for more details.

**Table 2:** Top 10 journals and co-cited journals on the research of NETs in RA.

Rank	Journals	Counts	IF (2023)	Co-Cited Journals	Counts	IF (2023)
1	Frontiers in immunology	46	8.786	Journal of immunology	312	5.426
2	International journal of molecular sciences	13	6.208	Annals of the rheumatic diseases	300	27.973
3	Autoimmunity reviews	13	17.39	PloS one	281	3.752
4	Arthritis & rheumatology	11	15.483	Science	271	63.714
5	Journal of immunology	9	5.426	Arthritis research & therapy	262	5.606
6	PloS one	7	3.752	Arthritis rheum-us	262	7.764
7	Journal of immunology research	6	4.493	Pans	256	12.779
8	Journal of autoimmunity	6	14.511	Frontiers in immunology	254	8.786
9	Clinical reviews in allergy & immunology	6	10.817	Science translational medicine	249	19.319
10	Autoimmunity	6	2.957	Nature medicine	239	87.241

**Figure 4:**
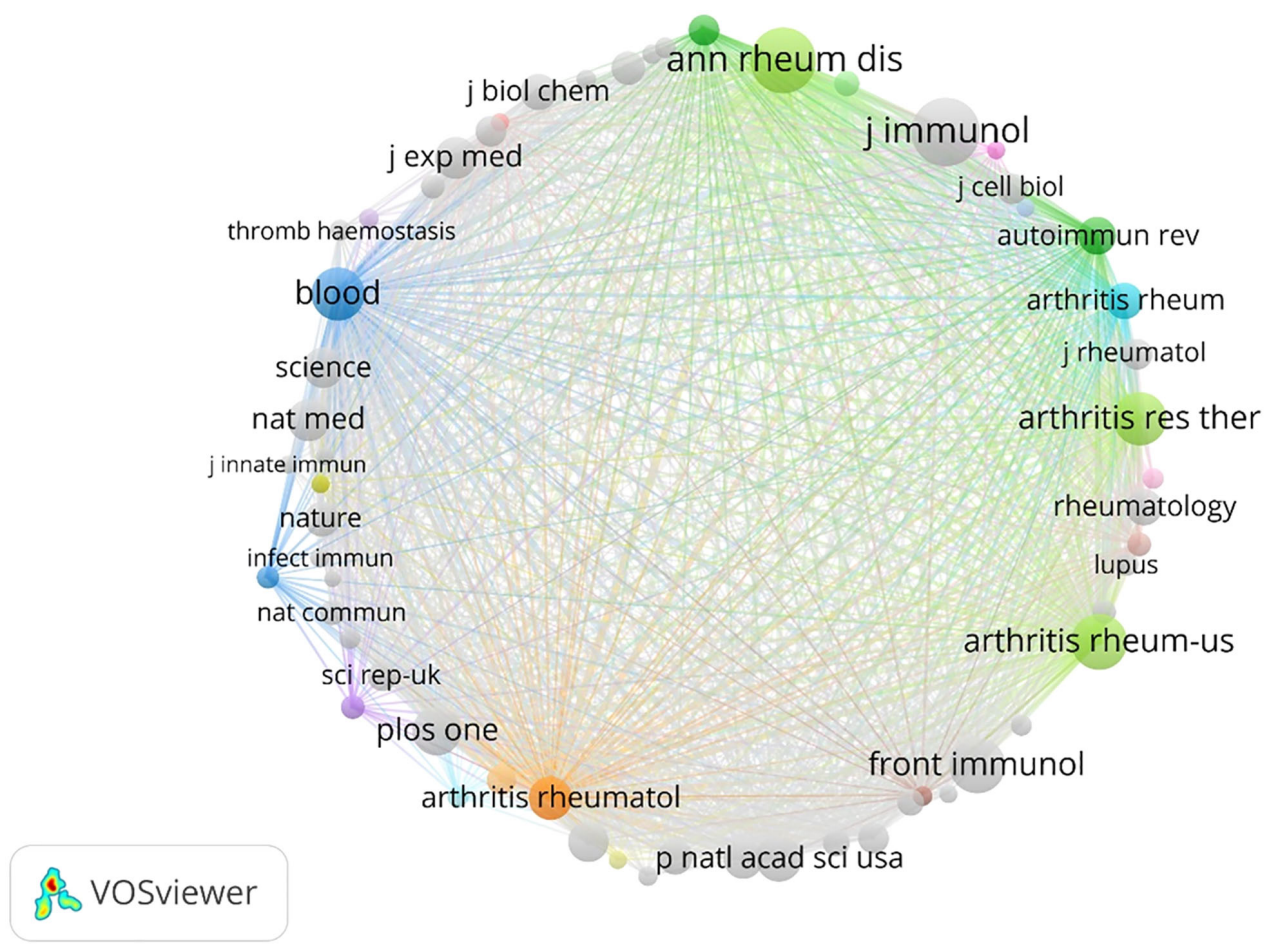
Visual representation of RA publications related to NETs in academic journals.

In this study, a total of 416 publications were analyzed, which were cited in 3564 journals. The co-citation analysis is presented in **Figure 4**, while the top 10 co-cited journals are listed in **Table 2**. Among these, the Journal of Immunology ranked first with 312 citations, followed closely by Annals of the Rheumatic Diseases with 300 citations, and PLoS ONE with 281 citations. Other notable journals in the top 10 included Arthritis Research & Therapy, which received 262 citations, and Science, which received 271 citations. It is worth noting that five of the top 10 co-cited journals had impact factors of 10 or higher in 2023, with the highest being Nature Medicine, which had an impact factor of 87.241.

### Analysis of top authors and co-cited authors

**Figure 5:**
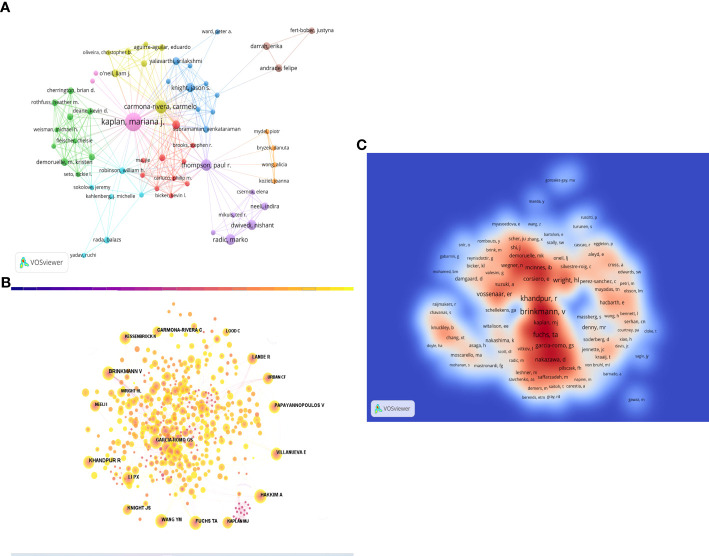
**(A)** Visual mapping of author collaborations associated with NETs in RA publications. **(B)** Visualization of co-cited Authors on the research of NETs in RA. **(C)** Total Cited Author Density Chart.

A total of 2,334 researchers have contributed to the study of NETs in RA over the past 40 years. **Figure 5A** shows the collaborative relationship between the authors. **Table 3** highlights the top 10 authors based on the number of publications, with Professor Mariana J Kaplan from the Division of Rheumatology, Department of Internal Medicine, University of Michigan Medical School, USA, ranking first with 18 publications. The Kaplan team has a remarkable research history on immune dysregulation in autoimmune diseases, including RA, and has extensively investigated the mechanisms of immune pathways that promote premature vascular injury in systemic autoimmunity, along with potential prevention strategies.

**Table 3:** Top 10 productive authors and co-cited authors.

Rank	Authors	Counts	Co-Cited Authors	Citations
1	Kaplan Mariana J	18	Brinkmann V	200
2	Carmona-Rivera, Carmelo	9	Khandpur R	180
3	Barbarroja Nuria	8	Fuchs TA	112
4	Radic Marko	5	Papayannopoulos V	105
5	Balthazart Jacques	5	Carmona-Rivera C	99
6	Luque-Tevar Maria	5	Hakkim A	98
7	Lopez-Pedrera Chary	5	Knight JS	91
8	Fox David A	5	Li PX	90
9	Perez-Sanchez Carlos	5	Villanueva E	88
10	Dwivedi Nishant	5	Wang YM	87

It is worth noting that Professor Carmelo Carmona-Rivera, who has the second-highest number of publications, is also from the Division of Rheumatology, Department of Internal Medicine, University of Michigan Medical School, suggesting the significant contributions of the Division of Rheumatology and Immunology at the University of Michigan to the development of this field. Jacques were ranked 3rd, 4th, and 5th with 8, 5, and 5 publications, respectively. Notably, three of the top five authors are from the USA, while the remaining two are from Europe.

In **Table 3**, the top 10 co-cited authors are also listed, along with the number of times they have been cited. BRINKMANN V from the Department of Neuroimmunology, Max-Planck-Institute of Neurobiology, Martinsried, Germany has been cited the most (200 times), followed by KHANDPUR R (180 times), FUCHS TA (112 times), PAPAYANNOPOULOS V (105 times), and CARMONA-RIVERA C (99 times). **Figure 5B** shows the mapping of author co-citation relationships, while the impact of co-cited authors in the field is demonstrated in density **Figure 5C**.

### Analysis of co-cited references and references citation bursts

**Figure 6:**
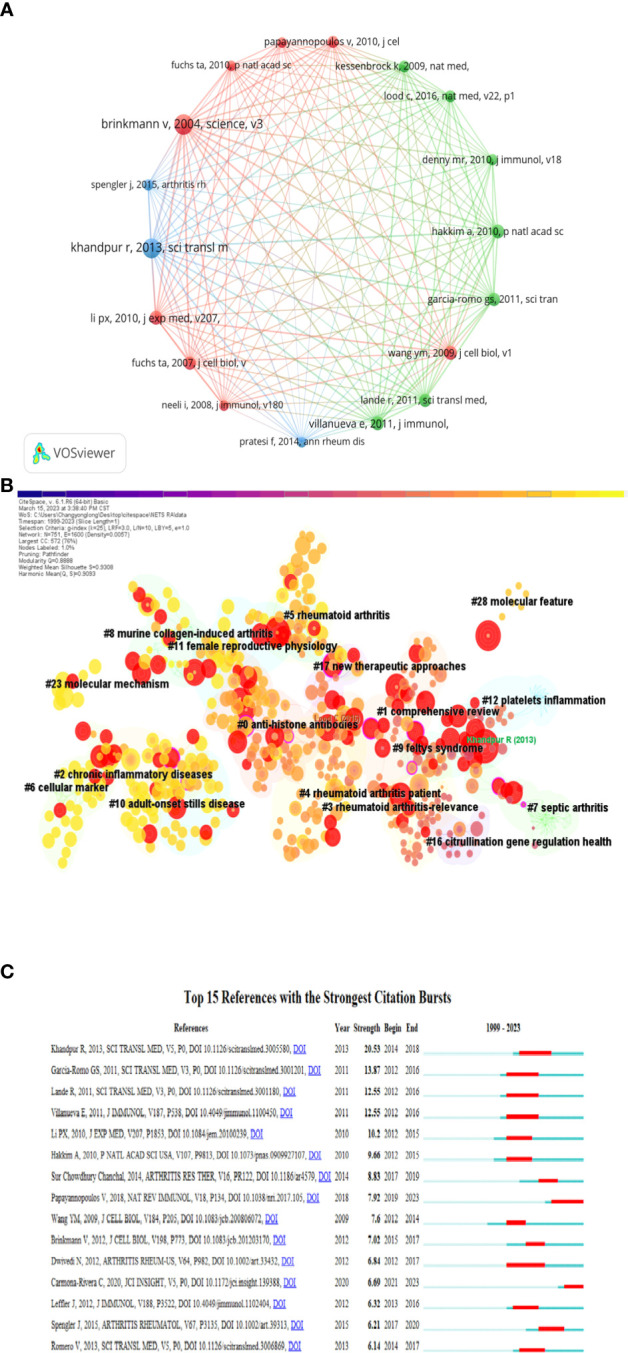
**(A)** A network map of co-cited publications with at least 50 citations. **(B)** Clustering chart of co-cited literature on NETs in RA research. **(C)** Top 15 outbreak literature by citation outbreak intensity.

When a publication is cited by two or more other publications simultaneously, it is known as a co-cited reference. The number of co-citations is an indicator of the importance of the literature in a particular field of study. In **Table 4**, Khandpur et al.’s 2013 article, titled “NETs are a source of citrullinated autoantigens and stimulate inflammatory responses in rheumatoid arthritis,” published in the journal Science Translational Medicine, ranked first with 81 citations. This study suggests that the formation of autoantigens and immunostimulatory molecules by NEs in RA may promote abnormal adaptive and innate immune responses in the joints and periphery, perpetuating the pathogenic mechanism of the disease. Additionally, the formation of autoantibodies to citrullinated protein antigens is a key pathogenic event in RA (4). **Table 4** provides details of the top 10 co-cited publications, their citation counts, and the country of their corresponding authors. Notably, nine corresponding authors are from both the Americas and Europe, reflecting the high quality of publications in this field from both regions. **Figure 6A** displays a network map of co-cited publications with at least 50 citations. Citespace clustering function was used to cluster the co-cited literature to explore the common themes of similar literature, as illustrated in **Figure 6B**. The most prominent cluster, ranked as #0, pertains to anti-histone antibodies, indicating its significance in the co-cited literature. Comprehensive review and chronic inflammatory diseases clusters are ranked #1 and #2, respectively, indicating their importance in the field as well.

**Table 4:** Top 10 co-cited publications.

Rank	Co-Cited Reference	Citations	Corresponding author’s country
1	Khandpur R, 2013, SCI TRANSL MED, V5, P0, DOI 10.1126/scitranslmed.3005580	81	USA
2	Lood C, 2016, NAT MED, V22, P146, DOI 10.1038/nm.4027	52	USA
3	Papayannopoulos V, 2018, NAT REV IMMUNOL, V18, P134, DOI 10.1038/nri.2017.105	43	England
4	Pratesi F, 2014, ANN RHEUM DIS, V73, P1414, DOI 10.1136/annrheumdis-2012-202765	42	Italy
5	Carmona-Rivera C, 2017, SCI IMMUNOL, V2, P0, DOI 10.1126/sciimmunol.aag3358	40	USA
6	Spengler J, 2015, ARTHRITIS RHEUMATOL, V67, P3135, DOI 10.1002/art.39313	37	England
7	Jorch SK, 2017, NAT MED, V23, P279, DOI 10.1038/nm.4294	35	Canada
8	Sur Chowdhury Chanchal, 2014, ARTHRITIS RES THER, V16, PR122, DOI 10.1186/ar4579	34	Switzerland
9	Lee KH, 2017, AUTOIMMUN REV, V16, P1160, DOI 10.1016/j.autrev.2017.09.012	34	Korea
10	Corsiero E, 2016, ANN RHEUM DIS, V75, P1866, DOI 10.1136/annrheumdis-2015-208356	32	England

The Burst detection algorithm, developed by Kleinberg, is a highly useful analytical tool that enables the capture of sudden surges in the popularity of keywords or references within a defined timeframe. This feature facilitates the rapid identification of concepts or topics that are currently being actively discussed within a particular period.

**Figure 6C** depicts the top 15 citation bursts in literature ranked by citation burst intensity. The earliest citation burst took place in 2012, and the latest occurred in 2021, with the longest burst spanning four years. The most recent burst is attributed to a review by Papayannopoulos V et al. from the Francis Crick Institute, UK, titled “Neutrophil extracellular traps in immunity and disease,” which was published in the journal Nature Reviews Immunology in 2018. This review offers a systematic analysis of the significant findings and concepts that have contributed to the field of NETs biology up to 2018 (20). Notably, this review on NETs is still undergoing a citation explosion phase.

### Analysis of keywords

**Figure 7:**
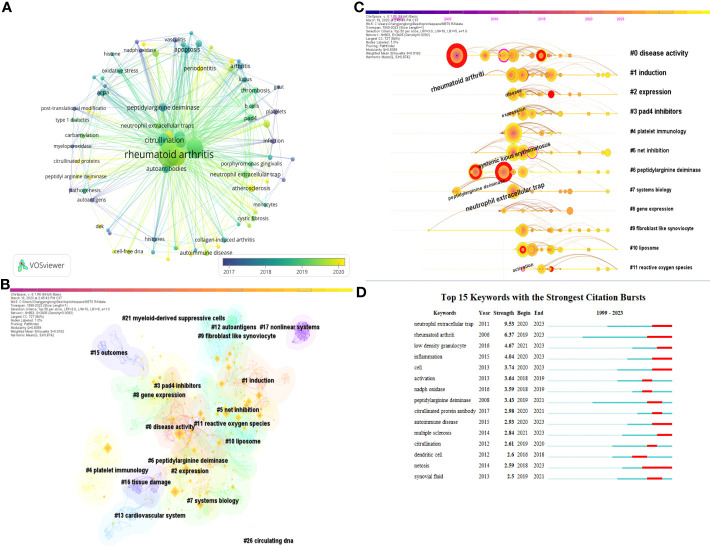
**(A)** a keyword co-occurrence network map. **(B)** Keyword co-occurrence clustering map. **(C)** Keyword co-occurrence clustering timeline diagram. **(D)** Top 10 keywords with the highest frequency.

Keywords serve as a brief summary of the article’s topic and analyzing them can provide insights into the current research status, research hotspots, and future directions of NETs in RA. **Figure 7A** presents a keyword co-occurrence network map, and the top 10 keywords with the highest frequency are displayed in **Table 5**. Notably, “rheumatoid arthritis,” “neutrophil extracellular trap,” and “systemic lupus erythematosus” ranked as the top three keywords with 263, 205, and 92 mentions, respectively, indicating that SLE, RA, and NETs are closely related research topics.

**Table 5:** Top 10 keywords with the highest frequency.

Rank	Keywords	Counts	Rank	Keywords	Counts
1	rheumatoid arthriti	263	6	disease	34
2	neutrophil extracellular trap	205	7	activation	34
3	systemic lupus erythematosus	92	8	netting neutrophil	32
4	peptidylarginine deiminase	41	9	autoimmune disease	32
5	expression	36	10	disease activity	32

After clustering all keywords, we created a clustering map, which is presented in **Figure 7B**. Of particular interest, peptidylarginine deiminase 4 (PAD4) inhibitors ranked fourth among the 21 clusters. As a member of the PAD family, PAD4 plays a critical role in the pathogenesis of RA, including in autoimmune response, inflammatory response, apoptosis, and other processes. It can generate new antigenic epitopes and activate immune responses. Inhibiting the activity of PAD4 may become a new strategy for treating RA by reducing the inflammatory and autoimmune responses and halting the progression of the disease.

Using the keyword clustering map, we generated a keyword timeline graph (**Figure 7C**), which highlights many keywords in the peptidylarginine deiminase clustering. This suggests that research on PAD and its inhibitors is a hot and frontier topic in this field. Additionally, research on reactive oxygen species (ROS) has emerged as a significant area in this field after 2015 and continues to be so. We also identified the top 15 most cited keywords (**Figure 7D**) and ranked them by citation intensity. This analysis showed that research topics have rapidly increased within a specific period of time. For instance, keywords such as citrullinated protein antibody suggest that research on citrullinated protein and its antibodies is a research hotspot in this field. Similarly, the keyword low density granulocyte indicates that low density granulocytes are a current focus of research in the study of NETs and RA.

## Discussion

In our research, we employed five scientific tools to create a bibliometric map and visualization of 416 publications on NETs in RA from 1985 to March 14, 2023. Our study systematically evaluates the present state of research and potential future research areas in the field of NETs in RA. To the best of our knowledge, this is the first bibliometric review, summary, and outlook of this field. We used quantitative, qualitative, and integrative analyses to identify trends and changes in research, which will be discussed in detail in the following sections, providing a comprehensive overview of our findings.

### General information

The analysis of the annual growth trend of publications in this field indicates a steady increase in the number of publications each year, with over 50 publications per year after 2018, and an R^2^ value of 0.914, suggesting that the research on NETs in RA is still in its early stages and has significant potential for further development. The US leads the world in the number of publications in this area, with 124 publications, largely due to the high productivity of the National Institute of Arthritis and Musculoskeletal and Skin Diseases (with 17 publications) in this field. China and Germany follow in second and third place with 57 and 37 publications, respectively. Although several partnerships have been established between countries in this research area, the US, China, and Italy have emerged as the most prominent collaborating nations. However, despite the success of these collaborations, **Figure 3B** suggests that many countries conduct research independently in this field, indicating the presence of academic barriers that impede cross-border cooperation among researchers. These obstacles could include uneven quality in research methodologies, insufficient funding or resources, and institutional or bureaucratic hurdles. By addressing these challenges, greater international collaboration and innovation can be fostered, ultimately advancing the field and contributing to scientific progress on a global scale.

In terms of authorship, Professor Mariana J. Kaplan, from the Department of Rheumatology and Department of Internal Medicine at the University of Michigan Medical School, USA, ranked first with 18 papers. Frontiers in Immunology is the most published journal in this field. Moreover, seven out of the top ten journals in this area have an impact factor of 5 or more, with four having impact factors of 10 or more, which is indicative of the high quality of research being conducted in this field.

### Knowledge base

After reviewing highly cited literature, a comprehensive understanding of NETs in RA can be established. Discovered in 2004 by Brinkmann et al., NETs are fibrous reticular structures composed of various immobilized protein particles that form through a process called NETosis (21). This process is an effective means by which neutrophils clear pathogens and can be initiated through two pathways, depending on whether it is mediated by NAPDH or not (9, 22, 23). The classical NADPH-mediated pathway involves the production of oxygen species ROS, which causes oxidative stress and impaired mitochondrial metabolism, followed by nuclear membrane rupture, a distinctive feature of NET formation. The second pathway, which is not mediated by NADPH, is facilitated by the opening of voltage-dependent calcium-activated potassium channel 3 (SK3) (24, 25). This leads to an increase in intracellular cytoplasmic calcium ion concentration and activates PAD4, causing chromatin decondensation (26). The decondensed chromatin then binds granzymes, such as NE and MPO, and other proteins before being released into the extracellular compartment, ultimately leading to the formation of NETs (27, 28).

The pathogenesis of rheumatoid arthritis (RA) is multifaceted, involving a complex interplay of genetic, environmental, and immune factors that are not fully understood. However, studies have shown that autoantibodies to Citrullinated protein (ACPA) and rheumatoid factor (RF) can be detected years before clinical diagnosis, with ACPA being highly specific for RA (29, 30). As a well-known autoantibody in RA, ACPA can recognize post-translational modification (PTM) citrullination, a process of immune tolerance, which is enzymatically catalyzed by peptidylarginine deiminase (PAD). The reaction converts arginine to citrulline. Neoantigens are generated during this process and become additional targets during epitope diffusion. Notably, ACPA and immune complexes containing citrullinated antigens are highly inflammatory and arthritic, correlating with disease severity. Candidate citrullinated autoantigens include vimentin, antithrombin, α-enolase, and fibrinogen, which have been suggested to be likely produced by peptidylarginine deiminase (PAD) 2 and 4 expressed in bone marrow cells, but *in vivo* evidence is less convincing because ACPA does not transfer arthritis compared with anti-collagen antibodies (31–33).

Neutrophils are present in increased numbers in the synovial fluid of RA patients and may contribute to joint injury and disease development, although their exact role in autoantigen modification and the persistence of RA is unclear (34). Histone guanylation catalyzed by PAD4 is a key step in NETosis, a process by which neutrophils release neutrophil extracellular traps (NETs) to capture and kill invading pathogens (35). Guanylated histones are externalized in NETs, which are enhanced in the synovial membrane of RA patients and contain autoantibodies targeting citrullination. This phenomenon is associated with the presence and level of ACPA antibodies and systemic inflammation (36).

While NETs play a crucial role in the non-specific immune response against pathogen invasion in the human body, they also act as a double-edged sword, potentially contributing to the development of RA. Therefore, multiple studies aim to demonstrate the therapeutic potential of modulating NETs to treat RA. Experimental animal research has indicated that targeting neutrophils can alleviate tissue inflammation and inhibit the progression of arthritis in the collagen antibody-induced (CIA) arthritis mouse model (37). Furthermore, Liu et al. discovered that downregulation of endothelial protein C receptor (EPCR) expression affects the disease progression in the CIA arthritis mouse model, which may be the pathway regulating PMN-NET secretion in RA patients (38). The role of NETs in RA involves PAD, ROS, nuclear factor kappa B (NF-κB), and ACPA, among other factors. Further research is needed to fully understand the mechanisms underlying the pathogenesis of RA and the role of NETs in this process.

### Emerging topics

Based on an analysis of highly cited literature and keywords in RA studies, it appears that there is a significant research focus on PAD enzymes and their inhibitors in relation to NETs. Additionally, multiple studies have mentioned ACPA as the primary diagnostic biomarker for early RA. Moving forward, there is a growing trend in developing new therapies to control the progression of NETs, which shows promise for future clinical applications.

PAD enzymes play a significant role in the pathogenesis of RA by promoting the formation of citrullinated proteins, which are the hallmark autoantigens of RA (39). Among the five isoforms of PAD enzymes, PAD2 and PAD4 are the most closely related to RA at the genetic and cellular levels (40, 41). Inhibition of PAD enzymes has shown therapeutic effects in mouse models of inflammatory arthritis (42). Furthermore, autoantibodies against PAD2 and PAD4 have been identified in different clinical subgroups of RA patients, suggesting that anti-PAD antibodies may serve as biomarkers for the diagnosis and prognosis of RA (43, 44). Overall, these findings suggest that targeting PAD enzymes may provide a new avenue for the treatment of RA. A case-control study was conducted by Spengler J et al. (45). in 2015 with 86 patients, and it was discovered that the levels of extracellular DNA in RA synovial fluid (SF) were significantly higher than those in SF from patients with osteoarthritis (OA) or psoriatic arthritis (PsA). The expression levels of extracellular DNA were found to be correlated with neutrophil concentrations and PAD activity in RA SF. The researchers also detected guanylated proteins PAD2 and PAD4 attached to NETs, which could diffuse freely in the supernatant. In addition, PAD enzyme activity was observed in the supernatant of neutrophils undergoing NETosis or necrosis. These findings indicate that neutrophil death leading to the release of active PAD isoforms into SF is a plausible explanation for extracellular autoantigen production in RA.

The formation of NETs correlated with the presence of ACPA and the levels of systemic inflammatory markers. Extracellular ACPA-induced release of PAD from neutrophils in RA, which catalyzes post-translational modification of arginine to citrulline and accelerates the progression of RA, also suggests that NETs are a source of ACPA implicated in the pathogenesis of RA (6, 46). A study conducted in 2013 by Khandpur R et al. (4). found that enhanced NETosis was observed in circulating and RA synovial neutrophils, compared to those from healthy controls and patients with OA. This increase in NETosis was found to be correlated with the presence and levels of ACPA, as well as systemic inflammatory markers. Further analysis showed that RA serum and immunoglobulin fractions from patients with high levels of ACPA and/or RF significantly enhanced NETosis. The NETs induced by these autoantibodies also showed varying protein levels, and were found to significantly enhance the inflammatory response of RA and OA synovial fibroblasts, including the induction of IL-6, IL-8, chemokines, and adhesion molecules (47). These findings suggest that accelerated NETosis plays a role in the pathogenesis of RA by promoting abnormal immune responses in the joint and periphery, thereby perpetuating the pathogenic mechanisms of the disease. Additionally, in 2013 Romero V et al. (48). introduced the concept of cellular hyperguanylation, which is the marked guanylation of various molecular weight proteins in RA synovial cells caused by immune-mediated membrane lysis pathways mediated by perforin and membrane attack complexes (MAC) that are activated in RA joints and subsequently produce ACPA (49).

Taken together, the existing studies suggest that the role of NETs in disease progression in RA is at least twofold. First, they produce some autoantigens associated with ACPA production and enhance the chance of autoantigen exposure to immune cells. Second, they release a variety of inflammatory factors to promote the development and maintenance of RA, which, as an autoimmune disease, is characterized by an unknown etiology, many complications, a high disability rate, and costly disease control (50–52). It is a highly burdensome type of disease. Since NETs were reported to be possibly related to autoimmune diseases, studies on their association with RA have been reported one after another, which has led to breakthroughs in the etiology and treatment of RA. A bibliometric study of the previously published literature revealed that NETs do not explain all the problems in RA. They may be only one link in the development of the RA disease, and there are still many questions to be further investigated (53). Based on the existing scientific knowledge map, we can speculate that the research and development of PAD enzymes and their inhibitors, the relationship between low-density neutrophils, NETosis, and ACPA, the relationship between NETs and ACPA, and the control of NETs as a therapeutic target for RA are the focus of future research in this field.

### Limitations

The utilization of visual analysis tools such as CiteSpace and VOSviewer allows for a more detailed understanding of changes in the structure and trends of NETs research junctions in RA. Nevertheless, this study has some limitations. First, the search was limited to publications in the core dataset of the Web of Science database and included only English-language literature, which may have led to the exclusion of some relevant studies. Therefore, the findings may not be comprehensive. In addition, there may be bias in the literature selection process.

## Conclusion

Based on the comprehensive analysis, it is evident that NETs play a crucial role in the development of RA. The number of publications related to NETs and RA has been steadily increasing year by year. The US, China, and Germany are among the leading countries that have made significant contributions to this area of research. Among the institutions with publications in this field, the NIAMS has the highest number of publications. Mariana J. Kaplan, from the Rheumatology Division of the University of Michigan, is the most published scholar with a long history of research on autoimmune diseases. The clinical application of PAD enzymes and their inhibitors, and the drug development of NETs as therapeutic targets for RA is a trend for future research.

## Data availability statement

The original contributions presented in the study are included in the article/supplementary material. Further inquiries can be directed to the corresponding author.

## Author contributions

YC and KN: Analyzing data and writing manuscripts. QO and JL: Screening Publications. XZ and SZ: Conceptualization and Writing - review & editing. All authors contributed to the article and approved the submitted version.
